# Conformity Assessment of Medical Devices: An Overview from a Notified Body

**DOI:** 10.3390/jmahp14010004

**Published:** 2026-01-08

**Authors:** Andreas F. Stange, Elaine Julian

**Affiliations:** 1 TÜV Süd, Osaka 542-0076, Japan; 2 Secretariat of the European Access Academy, 4059 Basel, Switzerland

**Keywords:** notified body, medical devices, *In Vitro* Diagnostics, IVD, MDR, conformity assessment

## Abstract

This perspective provides an in-depth analysis of the role and tasks of Notified Bodies (NBs) under the Medical Device Regulation (MDR) and *In Vitro* Diagnostic Regulation (IVDR). It explores the conformity assessment process and highlights typical issues encountered. It provides background on the legal framework and roles and tasks of Notified Bodies (NBs). It further explores the seven-step conformity assessment process which aims to ensure that medical devices meet European Union (EU) safety and performance standards. Finally, we highlight typical issues encountered during the process and re-cent developments in the area and conclude with an outlook for the implementation of the MDR and IVDR.

## 1. Introduction

The medical device industry is heavily regulated to ensure the safety and efficacy of products, with differing systems and internal regulations [1]. In the United States of America, the Food and Drug Administration (FDA) as a governmental institution is responsible for pre-market approvals for novel devices [2]. While this task is performed by closely supervised private third parties in the European Union (EU) and the United Kingdom [3], Japan chose a hybrid model: Low risk devices are certified by recognized certification bodies, while higher risk devices are approved by the Pharmaceutical and Medical Devices Agency [4]. In the EU, notified bodies (NBs) play a crucial role in this regulatory framework, particularly under the Medical Device Regulation (MDR) and *In Vitro* Diagnostic Regulation (IVDR) [5].

This perspective aims to provide a comprehensive understanding of the functions and responsibilities of NBs, the conformity assessment process, and common challenges faced during this process. The insights are based on a lecture “Comparative Evidence Generation for HTA—A NB perspective” by Dr. Andreas Stange, Senior Vice President Medical and Health Services at TÜV SÜD, held at a symposium by the European Access Academy at LUISS Business School, Rome, Italy on 24 October 2024.

## 2. Legal Framework

The legal framework for medical devices and *in vitro* diagnostic medical devices (IVDs) in Europe is primarily governed by Regulation (EU) 2017/745 on medical devices (MDR) and Regulation (EU) 2017/746 on *in vitro* diagnostic medical devices (IVDs) [6,7]. These regulations, which came into full application in May 2021 and May 2022, respectively, aim to ensure high standards of quality and safety for medical devices and IVDs. They introduce more stringent requirements for the conformity assessment, clinical evaluation, and post-market surveillance of these products. The European Medicines Agency (EMA) and national competent authorities play crucial roles in the implementation and oversight of these regulations, ensuring that medical devices and IVDs placed on the EU market are safe and perform as intended [8].

The Medical Device Coordination Group provides guidance documents to assist stakeholders in applying the MDR and IVDR [9]. These documents, while not legally binding, present a common understanding of how the regulations should be applied in practice, aiming for effective and harmonized implementation.

Additionally, the European Commission (COM) has extended the transition timelines for certain medical devices and IVDs to address challenges such as limited capacity of notified bodies and the need for more time to comply with the new regulations. The extended timelines allow for a staggered transition depending on the risk class of the devices, with deadlines extending to December 2027 for higher-risk devices and December 2028 for others [10].

## 3. Notified Body Roles and Tasks

NBs, such as TÜV SÜD, perform third-party assessments of medical device manufacturers’ quality management systems and products. As a result of such conformity assessments, EU certificates are issued, based on which manufacturers can declare conformity to applicable EU regulations and can subsequently place their products on the EU market. Based on specific designation requirements regarding organizational aspects, quality management, resources, and processes, as verified by relevant national authorities with the involvement of other European experts, NBs are “notified” by the COM. After notification they undergo annual surveillance by both domestic and EU authorities. NBs must adhere to a host of standards and rules set by the designation authority, thereby ensuring quality and safety. To date (May 2025), there are 50 and 17 NBs designated under the MDR and IVDR, respectively [11].

## 4. Personnel Qualifications

The resource requirements for NBs under the MDR and IVDR are laid out in Ax VII of the respective regulation [6,7]. As a result, personnel at NBs are highly qualified, with a university degree in medical or natural sciences. Additionally, they must possess over four years of professional experience in device technology, ensuring a deep understanding of their fields. Clinical experts are involved in evaluating clinical data, ensuring comprehensive assessments. Further, all NB staff are required to demonstrate professional integrity and have a deep understanding of quality management systems to effectively audit and assess manufacturers’ processes.

## 5. Conformity Assessment Activities

The conformity assessment involves three key activities to ensure medical devices meet EU safety and performance standards: auditing quality management Systems (QMS), assessing technical documentation, and testing devices. QMS audits are generally conducted on the premises of manufacturers after the system documentation has been reviewed. Expert reviews of technical documentation (TD) are performed off-site, often by several specialists like biocompatibility experts or clinicians. Testing is performed on devices sampled from manufacturers’ sites or from the market. Each of these steps is required to ensure compliance and quality.

## 6. MDR Certification Process

**Figure 1:**
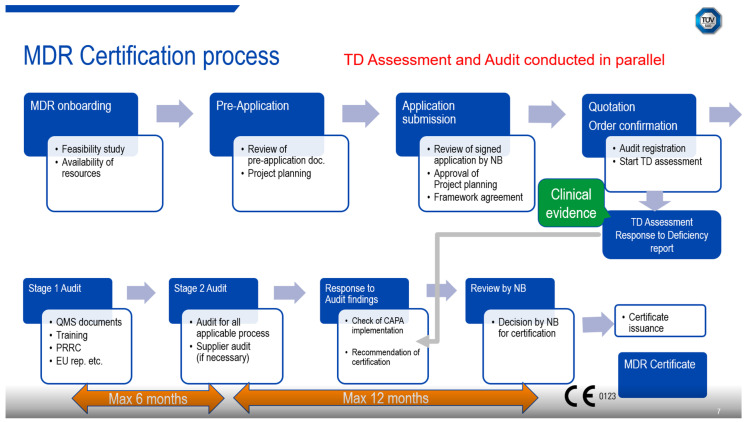
Overview of the MDR certification process. (Slide as shown at the inaugural EAA Roundtable EU HTA for Medical Devices, Rome, Italy, 24 October 2024.).

MDR certification is a comprehensive process, beginning with a pre-application review and application submission. It includes checking the availability of resources, project planning approval and providing quotations, framework agreements, and order confirmations. The process at an NB involves audit registration and technical documentation assessment, followed by stage 1 and stage 2 audits, and finally, after assessment of all identified corrective actions and clarifying any open questions, the issuance of the EU certificate (Figure 1).

1. Pre-Application Review: This initial stage involves reviewing the pre-application documentation submitted by the manufacturer.
2. Application Submission: The manufacturer submits the application, which is reviewed by an NB. The NB assesses the completeness and adequacy of the documentation to ensure the device in question is a medical device, to confirm the classification and that the technical documentation meets the regulatory requirements. The NB also checks the availability of resources and plans the project accordingly.
3. Technical Documentation Assessment: The NB assesses the TD to ensure it meets the requirements of Annex II and Annex III of the MDR/IVDR. Compliance to the general safety and performance requirements (GSPR) and, if applicable, common specifications (CS) will be scrutinized. This includes evaluating clinical evidence, cybersecurity measures, and other relevant aspects.
4. Auditing Quality Management System: The NB conducts audits of the manufacturer’s QMS to ensure it complies with the regulatory requirements. This includes assessing the control of critical suppliers and the contractual obligations of economic operators. The audits are usually conducted on-site at the manufacturer’s premises.
5. Testing Devices: The NB performs product testing to verify that the devices meet the required standards. This may involve electrical safety, functional safety, electromagnetic compatibility as well as cybersecurity testing, biocompatibility testing, and other testing activities.
6. Certification Decision: Based on the assessment and audit findings, the NB makes a decision on whether to issue an EU certificate. This involves reviewing the corrective and preventive actions (CAPA) implemented by the manufacturer and ensuring compliance with the regulatory requirements.
7. Renewal of Certificates: EU certificates have a maximum validity of five years and need to be renewed where the manufacturer intends to continuously place medical devices on the EU market. The renewal process should be initiated well in advance of the certificate expiry date, usually at least one year.

## 7. Typical Issues

During the certification process, typical issues that might arise include confirming device classification and code assignment in the application, ensuring all documents are included and easily searchable in the TD assessment, meeting MDR/IVDR requirements for clinical evidence, addressing cybersecurity concerns, and controlling critical suppliers and contractual obligations during audits.

The description of the intended purpose is often a point leading to lengthy discussions: It is a critical point because device classification, and with that the conformity assessment route, depends on it. Here precise wording is key: Does the intended patient group include children or not? Can lay people operate the medical device or only trained clinicians? Notified body reviews often reveal inconsistencies of the intended purpose in application documents, the instructions for use of the medical device and the clinical evidence provided. Clinical data must for instance cover the complete scope of the intended purpose and not only part of it.

It is common that safety-critical components or the device itself is purchased from or manufactured by third party suppliers. The manufacturer placing this device on the EU market must demonstrate control over these suppliers. Under the regime of the previous medical device directives, it was sufficient for those suppliers to hold an EC certificate covering the procured device. This practice is no longer permissible under the MDR/IVDR and the manufacturer must for instance be in possession of the complete TD to understand the manufacturing processes and material in detail.

While increased evidence requirements due to the MDR/IVDR have been known and discussed at length since the adoption of the regulations, many manufacturers are still facing challenges [12,13]. Critical issues regarding design or quality of clinical data can lead to significant delays of the certification process, as manufactures may need more time for design modifications or to gather new information.

## 8. Recent Developments

**Figure 2:**
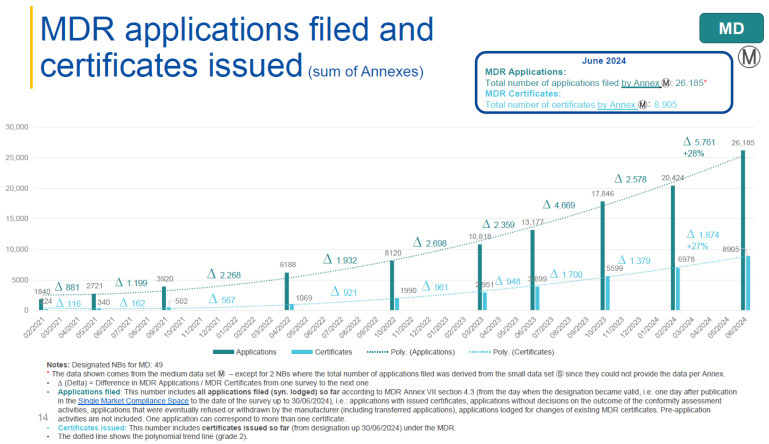
Development of MDR applications filed and MDR certificates issued by NBs, modified from [17].

The COM’s Directorate-General for Health and Food Safety has commissioned regular surveys on the availability of medical devices in Europe [14,15]. A dashboard is maintained and presents the data gathered from different stakeholders [16]. With more and more NBs being designated under both the MDR and IVDR the number of submissions by manufacturers for conformity assessments has been increasing as well: From the designation of the first MDR notified bodies in the first half of 2020 until summer 2023, approximately 13,000 applications have been received. In the following year, the number of applications doubled to more than 26,000. Likewise, the number of EU MDR certificates has been increasing at a steady pace from 3900 and 8900, respectively (Figure 2).

Despite the increasing numbers, the overall submission quality is still reported as being poor, 75% of notified bodies report that submissions are only 50% or less complete. This remains unchanged from previous surveys and is strong evidence that manufacturers still have difficulties with the implementation of regulatory requirements into their quality management systems.

**Figure 3:**
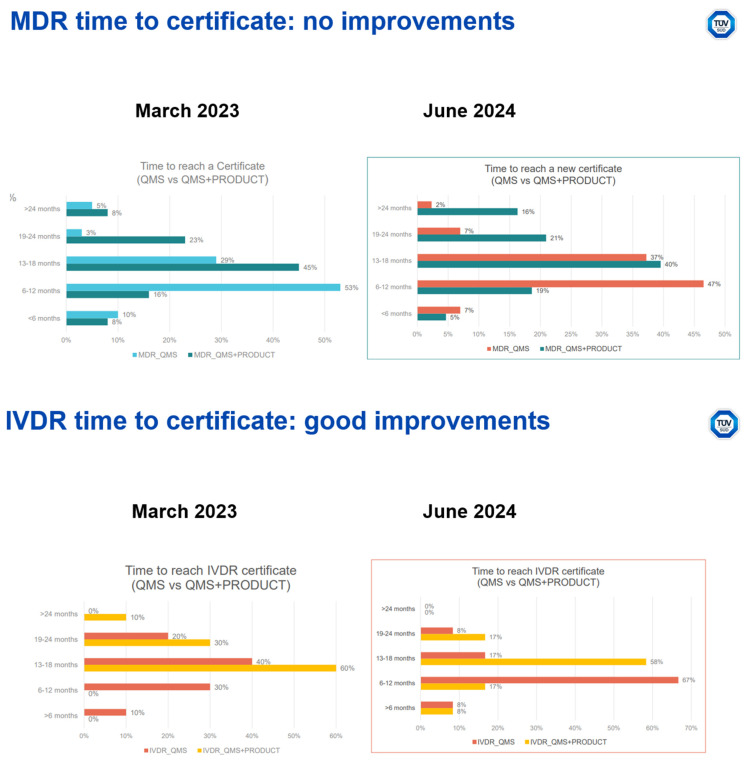
Development of submission quality, comparison of data from the 1st and 10th notified body survey on certifications and applications (MDR/IVDR) [14].

Poor submission quality is a key factor contributing to long turnaround times: Data collected in summer 2024 show MDR product certifications take in 75% of all cases more than a year, unchanged from a year before. IVDR product and QMS certification turnaround times show significant improvements in the same period, even though IVDR product certifications still need more than 12 months on average (Figure 3).

## 9. Conclusions

The implementation of MDR and IVDR is getting on track, which is evident from the increasing numbers of applications and certification. A serious concern remains with both submission quality of manufacturers and the time needed for conformity assessment processes conducted by notified bodies. Improved quality of the former will facilitate smoother and more predictable certifications. Both are needed to improve throughput and efficiency of the system and ensure patient safety for the European population also in future. It remains to be seen how and when the current rework of MDR and IVDR can contribute to this.

## Data Availability

The original contributions presented in this study are included in the article. Further inquiries can be directed to the corresponding author.
